# Elevated uric acid/albumin ratio as a predictor of poor coronary collateral circulation development in patients with non‐ST segment elevation myocardial infarction

**DOI:** 10.1002/clc.24215

**Published:** 2024-01-16

**Authors:** Renlin Yin, Zongwei Ye, Hua You, Yanming Wu, Weixiang Chen, Tingbo Jiang

**Affiliations:** ^1^ Department of Cardiology The First Affiliated Hospital of Soochow University Suzhou China; ^2^ Department of Cardiology Suzhou Ninth Hospital Affiliate to Soochow University Suzhou China

**Keywords:** coronary collateral circulation, non‐ST segment elevation myocardial infarction, Rentrop grade, uric acid to albumin ratio

## Abstract

**Background:**

Uric acid/albumin ratio (UAR) is a novel composite biomarker with superior predictive value for cardiovascular disease.

**Objective:**

To investigate the relationship between UAR and coronary collateral circulation (CCC) in patients with non‐ST segment elevation myocardial infarction (NSTEMI).

**Methods:**

A total of 205 NSTEMI patients who underwent coronary arteriography with at least one major coronary stenosis, 95% were included. Patients were divided into two groups according to CCC development: poorly‐developed CCC group (Rentrop 0–1) and well‐developed CCC (Rentrop 2–3). Univariate analysis and logistic regression analysis were utilized to investigate the factors influencing adverse CCC formation in NSTEMI patients. The receiver operating characteristic (ROC) curve was plotted to evaluate the predictive value of UAR, C‐reactive protein (CRP), uric acid, and albumin for patients with poorly developed CCC, and the area under the curve (AUC) was compared.

**Results:**

The UAR values of NSTEMI patients were significantly higher in the poorly developed CCC group than those in the well‐developed CCC group (10.19 [8.80–11.74] vs. 7.79 [6.28–9.55], *p* < .001). In the multiple logistic regression tests, UAR (odds ratio [OR]: 1.365, 95% confidence interval [CI]: 1.195–1.560, *p* < .001), CRP (OR: 1.149, 95% CI: 1.072–1.231, *p* < .001), and diabetes (OR: 2.924, 95% CI: 1.444–5.920, *p* = .003) were independent predictors of poorly developed CCC. The ROC curve analysis showed that the optimal cut‐off value of UAR was 8.78 in predicting poorly developed CCC with a sensitivity of 76.8% and specificity of 62.4%, with the AUC of 0.737 (95% Cl: 0.668–0.805, *p* < .001).

**Conclusion:**

Elevated UAR may be an independent and effective biomarker for predicting poorly‐developed CCC development in NSTEMI patients.

## Graphical abstract

Analysis of 205 non‐ST segment elevation myocardial infarction patients reveals the uric acid/albumin ratio (UAR) as a strong predictor of poor coronary collateral circulation development, outperforming UAR, C‐reactive protein, uric acid, and albumin levels in predictive value, as evidenced by receiver operating characteristic curves.

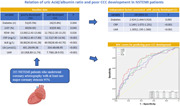

## 1 INTRODUCTION

Acute coronary syndrome (ACS) is a critical disease that poses a significant threat to public health. Non‐ST‐elevation myocardial infarction (NSTEMI) constitutes a substantial portion of patients affected by this syndrome.^1^ NSTEMI decreases myocardial blood flow, which, in turn, causes myocardial injury or infarction due to high coronary stenosis or complete interruption. Although these patients often exhibit insidious and atypical clinical symptoms, they frequently experience myocardial necrosis in extensive areas and have a poor prognosis.^2^ Coronary collateral circulation (CCC) formation represents an important protective adaptation of the body when the myocardium is severely ischemic. Well‐developed CCC provides partial relief to the ischemic myocardium, maintains ventricular function, and improves ischemic symptoms and hence the prognosis of patients with severe coronary artery disease (CAD). This phenomenon is often referred to as “natural coronary bypass.”

The formation of CCC is affected by various factors, including chronic myocardial ischemia, changes in blood flow pressure gradient, and the effect of inflammatory factors.^3^ In addition, comorbidities such as hypertension and diabetes, as well as physical exercise training and growth factors, have also been reported to influence CCC development.^4^, ^5^ C‐reactive protein (CRP) and red cell distribution width (RDW) have recently been reported to have a close relationship with CCC formation.^6^, ^7^ Uric acid (UA) is the end product of purine nucleotide catabolism. Hyperuricemia has been linked to the occurrence endothelial dysfunction, inflammation, oxidative stress,^8^, ^9^ increased morbidity, and poor prognosis in patients with coronary heart disease.^10^ Moreover, albumin (ALB) exhibits anti‐inflammatory, antioxidant, and antithrombotic effects, and low levels of ALB are associated with the development of various cardiovascular diseases.^11^ Therefore, identifying and understanding these factors is important to predict or even enhance the formation of CCC, especially in patients with compromised coronary blood flow.

The uric acid/albumin ratio (UAR) is a novel composite biomarker that provides superior predictive value for inflammation and oxidative stress compared to UA and ALB alone. UAR has demonstrated a strong independent predictive ability for the severity of CAD in NSTEMI patients.^12^ Studies have reported that UAR is significantly associated with no‐reflow in patients with ST‐elevation myocardial infarction (STEMI) undergoing primary percutaneous coronary intervention (PCI).^13^ Additionally, UAR has been identified as a predictor of high mortality in STEMI patients.^14^ Recently, a few studies showed that UAR was closely associated with CCC in stable CAD.^15^, ^16^ To date, no studies have investigated the association between UAR and CCC development in patients with NSTEMI. Therefore, the purpose of our study was to assess the relationship between UAR and CCC development in NSTEMI patients and discuss the potential value of UAR in assessing CCC formation.

## 2 METHODS

This retrospective study included 205 patients diagnosed with NSTEMI who consecutively underwent coronary angiography and were evaluated for CCC development in our catheterization laboratory between January 2018 and January 2023. The diagnosis of NSTEMI was based on the criteria recommended by the Fourth Universal Definition of Myocardial Infarction (2018).^17^ The inclusion criterion for this study was as follows: patients aged above 18 years with at least 95% diameter stenosis in at least one major epicardial coronary artery. Patients were excluded if they met any of the following criteria: previous history of ACS, percutaneous coronary angioplasty or coronary artery bypass surgery, severe myocardiopathy, pulmonary infarction, moderate to severe liver and renal dysfunction, hematological disease, autoimmune disease, malignancy, use of medicines that could affect UA levels, acute or chronic inflammatory disease, or insufficient data. The study was approved by the ethics committee of Suzhou Ninth Hospital affiliate to Soochow University (Decision no: KYLW2023‐010‐01), and conducted in accordance with the declaration of Helsinki. Written informed consent was obtained from all patients in this research.

Upon admission, the general data of patients were collected and assessed, including age, sex, smoking status, family history of CAD, the interval between the onset of pain and hospital admission, Killip status, previous medication and medical history of hypertension, diabetes, and dyslipidemia as defined in the previous guideline.^18^ All patients underwent transthoracic echocardiography, and the left ventricular ejection fraction (LVEF) was measured using the biplane Simpson method. Hematological indices, including MPV, RDW, platelet count, and monocyte count, were recorded using the Sysmex XN9000 auto hematology analyzer (Sysmex). Biochemical indices, including ALB, UA, CRP, and low‐density lipoprotein cholesterol (LDL‐C), were measured using the ADVIA2400 auto biochemical analyzer (Siemens). Blood samples were collected from patients after admission before the PCI.

Coronary angiography was performed within 48 h using the Judkins technique via the radial or femoral artery. Patients with at least one major coronary stenosis of 95% or greater were included. The angiographic images were evaluated by two experienced interventional cardiologists. CCC was graded using the Rentrop classification. In this classification, the scarcity of any collateral vessel was classified as Grade 0; filling inside branches through collateral vessels without visibility of the epicardial segment was classified as Grade 1; incomplete filling of the major epicardial artery via collateral circulation was classified as Grade 2; and complete filling of the major epicardial artery via collateral circulation was classified as Grade 3. For this study, patients were divided into two groups: Group 1 (well‐developed CCC: Grade 2–3) and Group 2 (poorly‐developed CCC: Grade 0–1). When multiple CCCs were present, the highest Rentrop grade was used for analysis. After PCI, an angiogram was performed to confirm the thrombolysis in myocardial infarction (TIMI) flow grade of the infarct‐related artery. Normal flow was defined as TIMI flow Grade 3, and slow flow/no‐reflow was defined as TIMI Grade 0–2 in the infarct‐related artery.

All statistical analyses were performed using SPSS version 24.0 (SPSS Inc.) for Windows. The distribution of quantitative variables was assessed using the Shapiro–Wilk test normally distributed variables were compared using the student *t*‐test and expressed as mean ± standard deviation. Non‐normally distributed variables were compared using the Mann–Whitney *U* test and expressed as median (interquartile range). Nonparametric variables between the two groups were compared using the chi‐square test. Variables with *p* < .10 in univariate testing and no significant correlation, as determined by Spearman's correlation analysis, were included in the multivariate regression analysis to identify independent predictors of poor CCC development. Receiver‐operating characteristic (ROC) analysis was used to estimate the predictive values of UA, ALB, CRP, and UAR. The area under the ROC curve (AUC) was compared using the Delong test in MedCalc statistical software version 20.0.22 (MedCalc Software Ltd.). A two‐sided *p*‐value < .05 was considered statistically significant.

## 3 RESULTS

A total of 205 patients with NSTEMI were enrolled in this study. Among them, 112 patients (mean age: 61.13 ± 13.11 years, male ratio: 73.2%) had poorly developed CCC, while 93 patients (mean age: 60.03 ± 13.79 years, male ratio: 75.3%) had well‐developed CCC. Table 1 presents the general characteristics and coronary angiographic data, showing that compared to patients with well‐developed CCC, those with poorly developed CCC had a higher frequency of diabetes (45.5% vs. 25.8%, *p* = .004) and lower LVEF (60 [56, 65] vs. 63 [59, 66], *p* = .040). There were no significant differences in other parameters between the two groups in Table 1.

**Table 1:** General characteristics and coronary angiographic data of the study populations.

Baseline variables	Poorly developed CCC (*n* = 112)	Well‐developed CCC (*n* = 93)	*t*/*z*/*χ* ^2^	*p*
Age (years, mean ± SD)	61.13 ± 13.11	60.03 ± 13.79	0.585	.559
men gender (*n*, %)	82 (73.2%)	70 (75.3%)	0.112	.738
Smoking (*n*, %)	63 (56.3%)	45 (48.4%)	1.260	.262
Hypertension (*n*, %)	64 (57.1%)	57 (61.3%)	0.361	.548
Diabetes (*n*, %)	51 (45.5%)	24 (25.8%)	8.525	.004
Dyslipidemia (*n*, %)	67 (59.8%)	51 (54.8%)	0.516	.472
Family history of CAD (*n*, %)	25 (22.3%)	23 (24.7%)	0.165	.685
the interval between the onset of pain and hospital admission (h, mean ± SD)	7.82 ± 4.96	7.37 ± 4.65	0.674	.501
Heart rate (I/min, mean ± SD)	78.78 ± 18.12	76.23 ± 13.40	1.157	.249
Systolic blood pressure (mmHg, mean ± SD)	128.16 ± 17.63	125.02 ± 20.26	1.186	.237
Diastolic blood pressure (mmHg, mean ± SD)	81.94 ± 13.51	79.67 ± 12.90	1.223	.223
LVEF [%, M (Q1,Q3)]	60 (56‐65)	63 (59‐66)	−2.052	.040
Killip status ( ≥ II) (*n*, %)	16 (14.3%)	9 (9.7%)	1.008	.315
Previous medication (*n*, %)				
Aspirin	31 (27.7%)	27 (29.0%)	0.046	.830
Insulin	15 (13.4%)	8 (8.6%)	1.171	.279
β‐blocker	21 (18.8%)	15 (16.1%)	0.241	.623
Statin	34 (30.4%)	32 (34.4%)	0.382	.537
ACE inhibitor/ARB	50 (44.6%)	49 (52.7%)	1.317	.251
Calcium channel blocker	39 (34.8%)	29 (31.2%)	0.303	.582
Number of diseased coronary vessels (*n*, %)				
1‐vessel disease	40 (35.7%)	30 (32.3%)	0.270	.603
LAD stenosis	18 (16.1%)	12 (12.9)	0.408	.523
LCX stenosis	12 (10.7%)	7 (7.5%)	0.614	.433
RCA stenosis	10 (8.9%)	11 (11.8%)	0.465	.496
2‐vessel disease	45 (40.2%)	39 (41.9%)	0.065	.799
LAD and LCX stenosis	19 (17.0%)	18 (19.4%)	0.196	.658
LAD and RCA stenosis	14 (12.5%)	10 (10.8%)	0.150	.698
LCX and RCA stenosis	12 (10.7%)	11 (11.8%)	0.063	.801
3‐vessel disease	27 (24.1%)	24 (25.8%)	0.079	.779
Postprocedural TIMI flow (*n*, %)				
III	101 (90.2%)	86 (92.5%)	0.334	.563
0–II	11 (9.8%)	7 (7.5%)		

Abbreviations: ACE, angiotensin‐converting enzyme; ARB, angiotensin receptor blocker; CAD, coronary artery disease; CCC, coronary collateral circulation; LAD, left anterior descending coronary artery; LCX, left circumflex coronary artery; LVEF, left ventricular ejection fraction; RCA, right coronary artery; TIMI, thrombolysis in myocardial infarction.

When comparing to patients with well‐developed CC, those with poorly‐developed CCC exhibited significantly higher levels of UA (401.26 ± 99.38 vs. 334.48 ± 98.95, *p* < .001), UAR (10.19 [8.80–11.74] vs. 7.79 [6.28–9.55], *p* < .001), CRP (14.98 [12.40–17.80] vs. 11.51 [7.90–14.94], *p* < .001), and RDW (13.00 [12.43–13.68] vs. 12.70 [12.30–13.25], *p* = .026). Conversely, ALB values were lower in poorly developed CCC groups compared to well‐developed CCC group (38.80 [36.83–41.38) vs. 40.90 [38.40–43.70], *p* < .001) (Table 2).

**Table 2:** Laboratory data of the study populations.

Parameter	Poorly developed CCC (*n* = 112)	Well‐developed CCC (*n* = 93)	*t*/*z*	*p*
WBC count (10^9^/L, M [Q1, Q3])	9.79 (7.85–11.68)	9.20 (6.88–11.54)	−1.251	.211
Platelet count (10^9^/L, mean ± SD)	248.79 ± 90.96	238.83 ± 62.48	0.896	.372
Mean platelet volume (fL, mean ± SD)	10.62 ± 1.43	10.48 ± 1.30	0.727	.468
Red cell distribution width (%,M [Q1, Q3])	13.00 (12.43–13.68)	12.70 (12.30–13.25)	−2.226	.026
Monocyte count (10^9^/L, mean ± SD)	0.56 ± 0.22	0.52 ± 0.26	1.161	.247
C‐reactive protein (mg/L, M [Q1, Q3])	14.98 (12.40–17.80)	11.51 (7.90–14.94)	−4.971	<.001
Neutrophil count (10^9^/L, M [Q1, Q3])	8.48 (6.44–10.64)	7.84 (6.06–9.94)	−1.363	.173
Lymphocyte count (10^9^/L, M [Q1, Q3])	1.44 (1.08–2.22)	1.53 (1.23–2.02)	−0.540	.589
ALT (U/L, mean ± SD)	21.03 ± 6.32	21.77 ± 6.54	−0.827	.409
AST (U/L, mean ± SD)	24.44 ± 7.17	23.61 ± 7.10	0.824	.411
LDL‐cholesterol (mmol/L, mean ± SD)	2.61 ± 0.85	2.49 ± 0.98	0.953	.342
HDL‐cholesterol (mmol/L, mean ± SD)	1.12 ± 0.34	1.07 ± 0.28	1.184	.238
Triglycerides (mmol/L, M [Q1, Q3])	1.25 (0.81–1.83)	1.17 (0.76–2.05)	−0.464	.643
Albumin (g/L, M [Q1, Q3])	38.80 (36.83–41.38)	40.90 (38.40–43.70)	−4.016	<.001
FBG (mmol/L, M [Q1, Q3])	5.16 (4.39–6.76)	5.02 (4.37–7.05)	−0.234	.815
Hemoglobin (g/L, mean ± SD)	139.29 ± 16.34	142.87 ± 15.47	−1.598	.112
Uric acid (umol/L, mean ± SD)	401.26 ± 99.38	334.48 ± 98.95	4.801	<.001
Baseline CK‐MB (U/L, M [Q1, Q3])	82.50 (21.25–134.75)	48.00 (20–112)	−1.340	.180
Creatinine (umol/L, mean ± SD)	79.11 ± 16.34	76.49 ± 14.44	1.204	.230
Uric acid/albumin (M [Q1, Q3])	10.19 (8.80–11.74)	7.79 (6.28‐9.55)	−5.827	<.001

Abbreviations: ALT, alanine aminotransferase; AST, aspartate aminotransferase; CCC, coronary collateral circulation; FBG, fasting blood glucose; HDL, high‐density lipoprotein; LDL, low‐density lipoprotein; WBC, white blood cell.

Univariate logistic regression analysis was performed to identify risk factors associated with CCC development from baseline clinical parameters based on a *p*‐value < .1 (Tables 1 and 2). These factors include diabetes (odds ratio [OR]: 2.404, 95% confidence interval [CI]: 1.326–4.358, *p* = .004), LVEF (OR: 0.961, 95%CI: 0.922–1.001, *p* = .058), CRP (OR: 1.153, 95%CI: 1.084–1.227, *p* < .001), RDW (OR: 1.386, 95%CI: 1.048–1.832, *p* = .022), ALB (OR: 0.851, 95%CI: 0.783–0.924, *p* < .001), UA (OR: 1.007, 95% CI: 1.004–1.010, *p* < .001), and UAR (OR: 1.383, 95%CI: 1.219–1.570, *p* < .001). Significant linear correlations were observed between UA and UAR (*r* = .938, *p* < .001) as well as between ALB and UAR (*r* = .373, *p* < .001). After eliminating UA and ALB, collinearity diagnostics were performed. Values of diabetes, RDW, LVEF, CRP, and UAR exhibited a variance inflation factor < 10, a tolerance degree > 0.1, and all of them were included in the multivariate logistic regression model. Multivariate logistic regression analysis showed that elevated UAR was an independent predictor of inadequate CCC (OR: 1.365, 95%CI: 1.195–1.560, *p* < .001), along with CRP (OR: 1.149, 95%CI: 1.072–1.231, *p* < .001) and diabetes (OR: 2.924, 95%CI: 1.444–5.920, *p* = .003). The Hosmer and Lemeshow test indicated that the model fit was good (*χ* ^2^ = 5.208 *df* = 8, *p* = .735) (Table 3).

**Table 3:** The results of univariate and multivariate Logistic regression analyses of influencing factors of poorly developed CCC.

Univariate analysis				Multivariate analysis		
Variables	Odds ratio	95%CI	*p*	Odds ratio	95%CI	*p*
Diabetes	2.404	1.326–4.358	.004	2.924	1.444–5.920	.003
LVEF	0.961	0.922–1.001	.058			
CRP	1.153	1.084–1.227	<.001	1.149	1.072–1.231	<.001
RDW	1.386	1.048–1.832	.022			
Albumin	0.851	0.783–0.924	<.001			
uric acid	1.007	1.004–1.010	<.001			
UAR	1.383	1.219–1.570	<.001	1.365	1.195–1.560	<.001

Abbreviations: CCC, coronary collateral circulation; CI, confidence interval; CRP, C‐reactive protein; LVEF, left ventricular ejection fraction; RDW, red cell distribution width; UAR, uric acid/albumin ratio.

**Figure 1:**
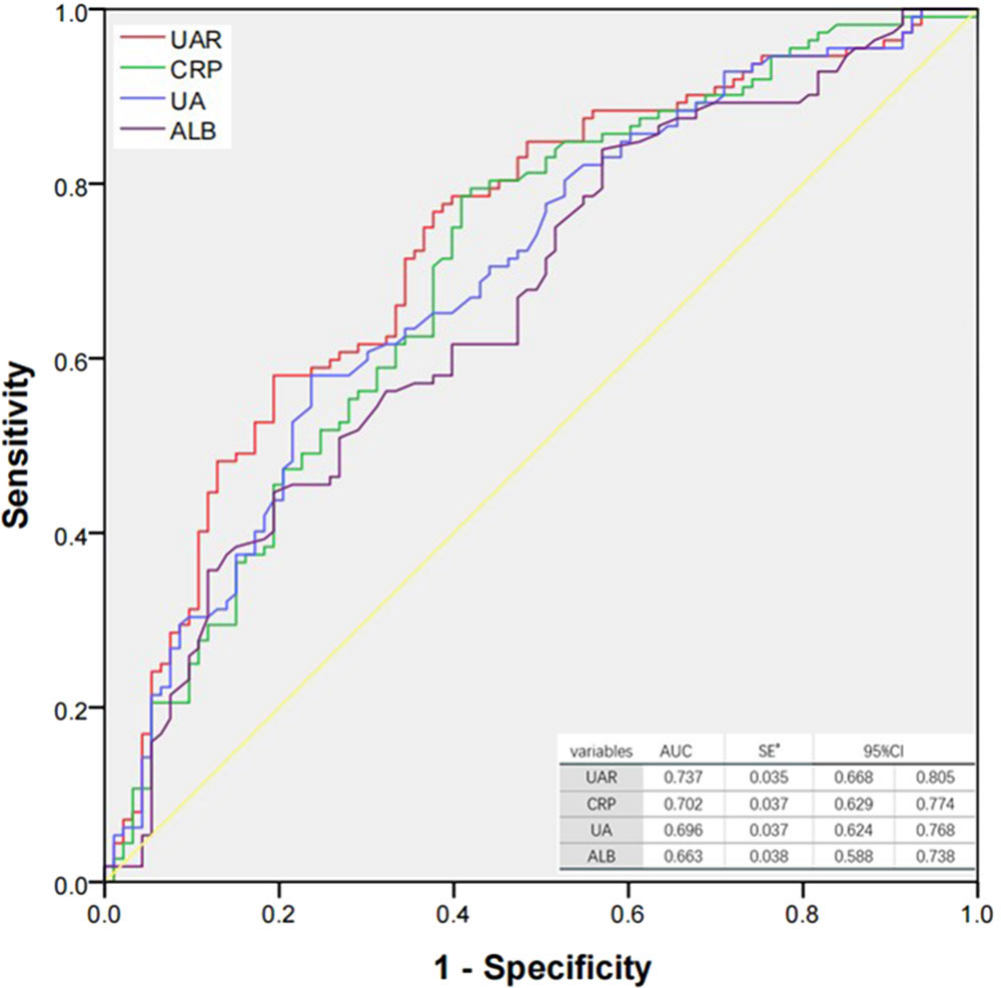
Receiver operating characteristic (ROC) curves for uric acid/albumin ratio (UAR), C‐reactive protein (CRP), uric acid (UA), and albumin (ALB) in predicting poor coronary collateral circulation.

The ROC curve analysis demonstrated that the cut‐off value of UAR for predicting poorly developed CCC was >8.78 with a 76.8% sensitivity and 62.4% specificity (AUC = 0.737, 95%CI: 0.668–0.805, *p* < .001). For CRP, a cutoff value > 12.21 indicated poorly developed CCC development, with 78.6% sensitivity and 59.1% specificity (AUC = 0.702, 95%CI: 0.629–0.774, *p* < .001). Additionally, a UA cut‐off value > 386.5 indicated poorly‐developed CCC with a 58% sensitivity and 76.3% specificity (AUC = 0.696, 95%CI: 0.624–0.768, *p* < .001). Furthermore, an ALB cutoff value > 42.37 demonstrated a sensitivity of 83.9% and specificity of 43.0% for prediction (AUC = 0.663, 95%CI:  0.588–0.738, *p* < .001) (Figure 1).

Results showed that UAR had higher accuracy than UA in predicting poorly‐developed CCC (UAR vs. UA: AUC: 0.737 vs. 0.696, *Z* = 3.090, *p* = .0020). Similarly, UAR also exhibited greater accuracy in predicting poorly developed CCC development compared to ALB (UAR vs. ALB: AUC: 0.737 vs. 0.663, *Z* = 2.996, *p* = .0027). However, there were no significant differences in accuracy for CCC prediction between UAR and CRP (UAR vs. CRP: AUC: 0.737 vs. 0.702, *Z* = 0.699, *p* = .4847) (Table 4).

**Table 4:** Comparison of the AUC of related indexes of UA, ALB, CRP, and UAR.

Variables	Difference between areas	Standard error	95%CI	*Z* statistic	*p*
UAR vs. UA	0.0408	0.0132	0.0149–0.0666	3.090	.0020
UAR vs. ALB	0.1270	0.0425	0.0441–0.211	2.996	.0027
UAR vs. CRP	0.0347	0.0497	−0.0627 to 0.132	0.699	.4847

Abbreviations: ALB, albumin; AUC, area under the curve; CRP, C‐reactive protein; UA, uric acid; UAR, uric acid/albumin ratio.

## 4 DISCUSSION

In our study, we demonstrated that NSTEMI patients with poorly developed CCC were more likely to have diabetes, higher UAR values and elevated CRP levels compared to those with well‐developed CCC. Our results indicated that UAR is a more effective independent predictor of poorly developed CCC than UA and ALB alone in patients with NSTEMI.

It is well known that the formation of CCC holds significant implications for patients with NSTEMI. Although the extent of coronary ischemia is similar, the degree of CCC formation varies significantly. CCC development may be affected by numerous factors. The role of inflammation and oxidative stress in CCC development is not clearly defined, but accumulating evidence suggests that inflammation and oxidative stress contribute to CCC development.^19^, ^20^, ^21^ Additionally, previous research has shown that various inflammatory parameters were associated with CCC formation and the prognosis of cardiovascular diseases, independent of traditional factors such as diabetes and renal insufficiency.^6^, ^7^ In our study, we found that diabetes, as a traditional risk factor, was independently associated with poorly developed CCC. Furthermore, CRP, a classic inflammatory maker, was significantly higher in patients with poorly developed CCC. These findings are consistent with those reported in previous research.^6^, ^20^ These associations are thought to be mediated by reduced production of NO and endothelial dysfunction. It is worth noting that while the AUC value of CRP in the ROC model was lower than that of UAR, there was no significant difference in the accuracy of prediction. Additionally, although high RDW was correlated with poorly‐developed CCC in the univariate analysis, it did not reach statistical significance in the multivariate analysis, which is inconsistent with previous studies in stable CAD patients.^7^ Previous investigations have reported that impaired renal function may be associated with poorly‐developed CCC in CAD patients,^19^, ^22^ but our study found no statistical differences between renal function and CCC formation. The discrepancies in research outcomes may be attributed to regional and ethnic variations across patient cohorts, as well as differences in the cardiac conditions studied.

UA is the end product of purine nucleotide catabolism in humans, originating from both endogenous and exogenous sources. Most studies, but not all of them, showed that elevated UA level was associated with various cardiovascular diseases and an increased risk of adverse cardiovascular outcome. The causal relationship between elevated UA levels and cardiovascular diseases remains controversial.^10^ However, several experimental studies have provided evidence supporting the role of UA in the pathophysiology of cardiovascular disease at the molecular and cellular level.^23^ Moreover, Kasapkar et al.^24^ demonstrated a significant association between high UA levels and poorly‐developed CCC development in NSTEMI patients. Uysal et al. provided evidence suggesting a negative association between high UA levels and CCC formation in patients with stable CAD.^25^ Duran et al.^26^ found that elevated UA levels prevent CCC formation in patients with ACS. Consistent with these findings, our study also suggested that higher UA levels were detrimental to CCC development. The association between UA and CCC development may be attributed to various mechanisms. Primarily, numerous studies have consistently suggested that UA can reduce NO production, thereby inducing endothelial dysfunction.^9^, ^10^ Several studies have also speculated that high UA levels may exert harmful effects on microvascular vessels.^27^ Additionally, UA might trigger inflammation leading to the release of vasoconstrictive substances and impaired endothelial function.^9^, ^10^, ^28^ Moreover, while there is an oxidant‐antioxidant paradox of UA, evidence suggests that UA can act as an antioxidant (mainly in the plasma) or as a pro‐oxidant (mostly within cells).^29^ Sautin and Johnson^30^ suggested that UA functions as an antioxidant only in a hydrophilic environment, which likely limits its antioxidant function. Yu et al.^8^ also found that oxidative stress is a mechanism of UA‐induced endothelial dysfunction.

ALB is an essential protein that transports and binds various ions, lipids, and metabolites in the body. It maintains colloid osmotic pressure and regulates the circulatory system. Hypoproteinemia has been associated with various cardiovascular diseases, including chronic and acute coronary syndrome, heart failure, hypertension, atrial fibrillation, ischemic stroke, and peripheral artery diseases.^11^, ^31^, ^32^, ^33^, ^34^, ^35^ In recent years, hypoproteinemia has been recognized as an underestimated predictor of cardiovascular disease. In our study, we found that decreased ALB was detrimental to CCC development which has also been shown in some studies.^15^ Mechanisms may explain this association as below. On the one hand, ALB is a negative acute‐phase reactant, and a deficiency in ALB levels contributes to increased inflammation. The antioxidant properties of ALB in cardiovascular disease have been well‐established.^31^ On the other hand, serum ALB also exhibits anticoagulant and antithrombotic/antiplatelet aggregation activity, which is beneficial for circulatory homeostasis and CCC development.^36^, ^37^

Despite the strong association of UA and ALB levels with cardiovascular diseases, supported by previous scientific evidence, there exist some controversies regarding the factors that affect UA and ALB concentration such as malnutrition, insulin, gout, diet, renal diseases, and metabolic syndrome.^38^ Taking these factors into consideration, we aimed to evaluate the relationship between UAR, a novel compositive biomarker, and CCC formation. UAR has been reported to be highly associated with the extent of CAD in NSTEMI patients.^12^ It has also been proposed to be a predictor of high mortality in patients with STEMI.^14^ Li et al. demonstrated a positive correlation between UAR and long‐term cardiac death in patients with unstable angina pectoris after PCI.^39^ The UAR index was associated with the occurrence of atrial fibrillation and kidney injury.^40^, ^41^ Furthermore, Toprak et al.^15^ and Şaylık et al.^16^ have found that UAR was a superior marker in predicting poorly‐developed CCC in stable coronary heart disease. However, the relationship between the UAR and CCC development in NSTEMI patients remains poorly understood. In light of our study, we confirmed that UAR exhibited a stronger predictive value for poorly developed CCC in patients with NSTEMI compared with UA and ALB alone. As previously discussed, elevated UA and decreased ALB form the mechanisms underlying the aggravation of poorly developed CCC and the cardiovascular system. Given the important role of CCC in NSTEMI patients, UAR may be a novel biomarker that will assist physicians in predicting poorly developed CCC in these patients.

## 5 LIMITATIONS

Firstly, this study is a single‐center retrospective study with a small sample size. Secondly, the study relied on medical records for collecting patient data, and certain important information, such as the history of physical exercise, could not be accurately provided. Thirdly, we only collected blood data in the early days of hospital admission, and the changes in these values during hospitalization were not assessed. Fourthly, several factors that may participate in CCC development, such as NO and VEGF, were not measured in this study. Lastly, the use of Rentrop classification as a method to assess CCC development has limitations, particularly in its ability to evaluate the development of small, microscopic vessels.

## 6 CONCLUSION

UAR, as an easily accessible, inexpensive, and conventionally tested biomarker, shows promise as an effective predictor for poorly‐developed CCC in patients with NSTEMI.

## CONFLICT OF INTEREST STATEMENT

The authors declare no conflict of interest.

## Data Availability

The data that support the findings of this study are available from the corresponding author upon reasonable request.
